# (*RS*)-1-(1-Acetyl­indolin-5-yl)-2-chloro­propan-1-one

**DOI:** 10.1107/S1600536810020969

**Published:** 2010-06-09

**Authors:** Xue-Mei Yang

**Affiliations:** a Department of Chemistry, Guangdong Medical College, Dongguan 523808, People’s Republic of China

## Abstract

The mol­ecule of the title compound, C_13_H_14_ClNO_2_, is roughly planar [maximum deviation = 0.060 (2) Å] with the disordered Cl/CH_3_ group asymetrically distributed on both sides of the mean plane. Indeed, the Cl and CH_3_ located on the stereogenic carbon exchange each other with occupancy factors in the ratio 0.60:0.40. The whole crystal is a racemate. Non-classical C—H⋯O hydrogen bonds and π–π inter­actions [centroid–centroid distance = 3.6959 (9) Å] between symmetry-related phenyl rings stabilize the crystal structure.

## Related literature

The title compound was synthesised as an inter­mediate in a search for a new synthetic route for silodosin, an adrenoceptor antagonist, see: Asselin *et al.* (2000 ▶); Bremner *et al.* (2000 ▶); Elworthy *et al.* (1997 ▶); Sorbera *et al.* (2001 ▶). For related structures, see: Moreno *et al.* (1998 ▶); Wang *et al.* (2007 ▶).
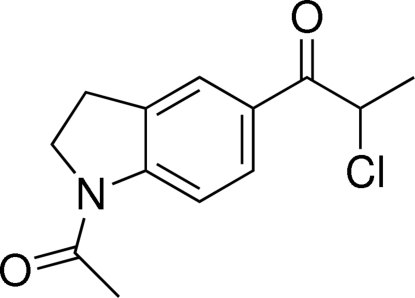


## Experimental

### 

#### Crystal data

C_13_H_14_ClNO_2_
*M* *_r_* = 251.70
Triclinic, 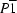

*a* = 8.4748 (5) Å
*b* = 9.0928 (5) Å
*c* = 9.4952 (5) Å
α = 112.071 (1)°
β = 110.345 (1)°
γ = 99.913 (1)°
*V* = 595.92 (6) Å^3^
*Z* = 2
Mo *K*α radiation
μ = 0.31 mm^−1^
*T* = 173 K
0.46 × 0.36 × 0.15 mm

#### Data collection

Bruker SMART 1000 CCD diffractometer
Absorption correction: multi-scan (*SADABS*; Sheldrick, 2008*a* ▶) *T*_min_ = 0.871, *T*_max_ = 0.955
6682 measured reflections
2594 independent reflections
2242 reflections with *I* > 2σ(*I*)
*R*_int_ = 0.018

#### Refinement

*R*[*F* ^2^ > 2σ(*F* ^2^)] = 0.043
*wR*(*F* ^2^) = 0.111
*S* = 1.18
2594 reflections
178 parameters
3 restraints
H-atom parameters constrained
Δρ_max_ = 0.27 e Å^−3^
Δρ_min_ = −0.21 e Å^−3^

### 

Data collection: *SMART* (Bruker, 2001 ▶); cell refinement: *SAINT-Plus* (Bruker, 2003 ▶); data reduction: *SAINT-Plus*; program(s) used to solve structure: *SHELXTL* (Sheldrick, 2008*b* ▶); program(s) used to refine structure: *SHELXL97* (Sheldrick, 2008 ▶); molecular graphics: *ORTEPIII* (Burnett & Johnson, 1996 ▶), *ORTEP-3 for Windows* (Farrugia, 1997 ▶) and *PLATON* (Spek, 2009 ▶); software used to prepare material for publication: *SHELXL97*.

## Supplementary Material

Crystal structure: contains datablocks global, I. DOI: 10.1107/S1600536810020969/dn2565sup1.cif

Structure factors: contains datablocks I. DOI: 10.1107/S1600536810020969/dn2565Isup2.hkl

Additional supplementary materials:  crystallographic information; 3D view; checkCIF report

## Figures and Tables

**Table 1:** Hydrogen-bond geometry (Å, °)

*D*—H⋯*A*	*D*—H	H⋯*A*	*D*⋯*A*	*D*—H⋯*A*
C1—H1*B*⋯O2^i^	0.99	2.44	3.252 (3)	139
C4—H4⋯O1^ii^	0.95	2.48	3.430 (2)	177
C12—H12⋯O1^ii^	1.00	2.41	3.318 (2)	151

Symmetry codes: (i) 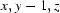; (ii) 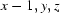.

**Fig. 1.**
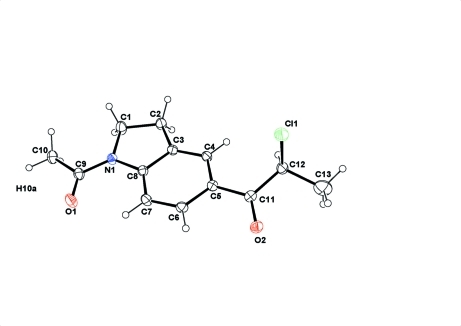
The asymmetric unit of (I) with the atom-labelling scheme. Displacement ellipsoids are drawn at the 30% probability level. H atoms are represented as small spheres of arbitrary radii. For clarity, only the major component of the disorder is represented.

**Fig. 2.**
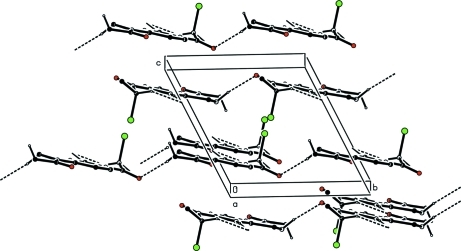
Packing view showing the layers formed by C—H···O interaction. Hydrogen bonds are shown as dashed lines. H atoms not involved in hydrogen bondings have been omitted for clarity.

## supplementary crystallographic information

### Comment

In searching for new synthetic route of silodosin, a adrenoceptor antagonist (Sorbera *et al.* 2001; Elworthy *et al.* 1997; Asselin *et al.* 2000; Bremner *et al.* 2000), we synthesized the racemic intermediate,(*R*/*S*)-1-(1-acetylindolin-5-yl)-2-chloropropan-1-one.

The single-crystal structure analysis shows that the Cl and CH_3_ located on the stereogenic carbon exchange each other with occupancy factor in the ration 60/40. Except for these disordered atoms, the molecule is roughly planar with the largest deviation from the mean plane (all heavy atoms except Cl and C13) being 0.060 (2)Å at C7 (Fig. 1). The two disordered atoms are dissymetrically distributed on both side of the mean plane. The geometry within the 1-acetylindoline fragment compares well with related structures as 1-acetylindoline (Moreno *et al.*, 1998) or 1-(trifluoro)acetylindoline (Wang *et al.*, 2007).

Non-classical C—H···O hydrogen bonds (Table 1, Fig. 2) link the molecules forming layers parallel to the (0 0 1) plane. These layers are further connected throught π-π interactions between symmetry related phenyl rings (Table 2).

### Experimental

3.3 g aluminium trichloride was added to 20 ml dichloromethane, and stirred for 10 min. Then 2 g chloropropionylchloride was added, controling the temperature below 5¯C. A dichloromethane solution of 1-acetyl-indoline was added dropwise to the reaction solution, and stirred overnight to get 1.3 g crystalline solid (yield 72%). Crystals suitable for X-ray diffraction were obtained by slow evaporation of an ethyl acetate solution. Spectroscopic analysis: ^1^H NMR (CDCl_3_,δ, p.p.m.): 1.723–1.760(d, 3H), 2.259–2.269(s, 3H), 3.232–3.289(t, 2H), 4.109–4.166(t, 2H), 5.197–5.263(m, 1H), 7.864(s,1*H*), 7.864–7.895(d, 1H), 8.245–8.273(d, 1H).

### Refinement

All H atoms attached to C atoms and N atom were fixed geometrically and treated as riding with C—H = 0.98 Å (methyl), 0.99 Å (methylene) and 1.0 Å (methine) with *U*_iso_(H) = 1.2*U*_eq_(Cmethine, Cmethylene) or *U*_iso_(H) = 1.5*U*_eq_(Cmethyl).

The Cl and CH3 substituents on the stereogenic carbon are exchanging each other and such disorder induces two configurations. Two sets of positions were defined for the atoms of this group and the site occupation factor of each conformation were refined while restraining their sum to unity and using restraints on C—C and C—Cl distances with the help of SAME and PART instructions within *SHELXL97* (Sheldrick, 2008). In the last stage of refinement, the disordered Cl and C atoms were anisotropically refined but the anistropic thermal parameters of the C atoms were restrained to have similar atomic displacement parameters within a tolerance s.u. of 0.01 Å^2^.

### Crystal data

**Table:**

C_13_H_14_ClNO_2_	*Z* = 2
*M**_r_* = 251.70	*F*(000) = 264
Triclinic, *P*1	*D*_x_ = 1.403 Mg m^−^^3^
Hall symbol: -P 1	Mo *K*α radiation, λ = 0.71073 Å
*a* = 8.4748 (5) Å	Cell parameters from 4108 reflections
*b* = 9.0928 (5) Å	θ = 2.6–27.0°
*c* = 9.4952 (5) Å	µ = 0.31 mm^−^^1^
α = 112.071 (1)°	*T* = 173 K
β = 110.345 (1)°	Block, colorless
γ = 99.913 (1)°	0.46 × 0.36 × 0.15 mm
*V* = 595.92 (6) Å^3^	

### Data collection

**Table:**

Bruker SMART 1000 CCD diffractometer	2594 independent reflections
Radiation source: fine-focus sealed tube	2242 reflections with *I* > 2σ(*I*)
graphite	*R*_int_ = 0.018
ω scans	θ_max_ = 27.1°, θ_min_ = 2.6°
Absorption correction: multi-scan (*SADABS*; Sheldrick, 2008a)	*h* = −10→10
*T*_min_ = 0.871, *T*_max_ = 0.955	*k* = −11→11
6682 measured reflections	*l* = −12→12

### Refinement

**Table:**

Refinement on *F*^2^	Primary atom site location: structure-invariant direct methods
Least-squares matrix: full	Secondary atom site location: difference Fourier map
*R*[*F*^2^ > 2σ(*F*^2^)] = 0.043	Hydrogen site location: inferred from neighbouring sites
*wR*(*F*^2^) = 0.111	H-atom parameters constrained
*S* = 1.18	*w* = 1/[σ^2^(*F*_o_^2^) + (0.0322*P*)^2^ + 0.3925*P*] where *P* = (*F*_o_^2^ + 2*F*_c_^2^)/3
2594 reflections	(Δ/σ)_max_ = 0.001
178 parameters	Δρ_max_ = 0.27 e Å^−^^3^
3 restraints	Δρ_min_ = −0.20 e Å^−^^3^

### Special details

**Table:**

Geometry. All e.s.d.'s (except the e.s.d. in the dihedral angle between two l.s. planes) are estimated using the full covariance matrix. The cell e.s.d.'s are taken into account individually in the estimation of e.s.d.'s in distances, angles and torsion angles; correlations between e.s.d.'s in cell parameters are only used when they are defined by crystal symmetry. An approximate (isotropic) treatment of cell e.s.d.'s is used for estimating e.s.d.'s involving l.s. planes.
Refinement. Refinement of *F*^2^ against ALL reflections. The weighted *R*-factor *wR* and goodness of fit *S* are based on *F*^2^, conventional *R*-factors *R* are based on *F*, with *F* set to zero for negative *F*^2^. The threshold expression of *F*^2^ > σ(*F*^2^) is used only for calculating *R*-factors(gt) *etc*. and is not relevant to the choice of reflections for refinement. *R*-factors based on *F*^2^ are statistically about twice as large as those based on *F*, and *R*- factors based on ALL data will be even larger.

### Fractional atomic coordinates and isotropic or equivalent isotropic displacement parameters (Å^2^)

**Table:**

	*x*	*y*	*z*	*U*_iso_*/*U*_eq_	Occ. (<1)
O1	0.9779 (2)	−0.01243 (19)	0.2587 (2)	0.0410 (4)	
O2	0.35372 (18)	0.39091 (17)	0.09272 (18)	0.0348 (3)	
N1	0.7298 (2)	−0.10127 (19)	0.28713 (19)	0.0271 (3)	
C1	0.6190 (3)	−0.2154 (3)	0.3214 (3)	0.0394 (5)	
H1A	0.6802	−0.1916	0.4415	0.047*	
H1B	0.5956	−0.3354	0.2463	0.047*	
C2	0.4441 (3)	−0.1788 (2)	0.2847 (3)	0.0323 (4)	
H2A	0.3427	−0.2765	0.1824	0.039*	
H2B	0.4166	−0.1526	0.3827	0.039*	
C3	0.4790 (2)	−0.0270 (2)	0.2553 (2)	0.0252 (4)	
C4	0.3712 (2)	0.0667 (2)	0.2265 (2)	0.0253 (4)	
H4	0.2594	0.0427	0.2300	0.030*	
C5	0.4281 (2)	0.1977 (2)	0.1918 (2)	0.0249 (4)	
C6	0.5941 (2)	0.2322 (2)	0.1900 (2)	0.0279 (4)	
H6	0.6319	0.3208	0.1661	0.033*	
C7	0.7055 (2)	0.1416 (2)	0.2219 (2)	0.0292 (4)	
H7	0.8188	0.1677	0.2217	0.035*	
C8	0.6455 (2)	0.0109 (2)	0.2544 (2)	0.0245 (4)	
C9	0.8883 (2)	−0.1095 (2)	0.2860 (2)	0.0296 (4)	
C10	0.9462 (3)	−0.2455 (3)	0.3189 (3)	0.0358 (4)	
H10A	0.8635	−0.3568	0.2239	0.054*	
H10B	0.9452	−0.2369	0.4246	0.054*	
H10C	1.0679	−0.2313	0.3298	0.054*	
C11	0.3175 (2)	0.3004 (2)	0.1525 (2)	0.0264 (4)	
C12	0.1597 (3)	0.2956 (2)	0.1952 (2)	0.0306 (4)	
H12	0.1025	0.1787	0.1740	0.037*	
C13	0.0115 (16)	0.3460 (17)	0.0833 (16)	0.058 (4)	0.60
H13A	−0.0826	0.3497	0.1206	0.087*	0.60
H13B	−0.0414	0.2617	−0.0370	0.087*	0.60
H13C	0.0678	0.4576	0.0975	0.087*	0.60
Cl1	0.2419 (3)	0.4356 (3)	0.40972 (18)	0.0464 (6)	0.60
C13B	0.244 (2)	0.4216 (19)	0.4003 (16)	0.072 (6)	0.40
H13D	0.3329	0.3851	0.4631	0.108*	0.40
H13E	0.1470	0.4173	0.4347	0.108*	0.40
H13F	0.3008	0.5379	0.4258	0.108*	0.40
Cl1B	0.0086 (5)	0.3588 (5)	0.0840 (4)	0.0361 (9)	0.40

### Atomic displacement parameters (Å^2^)

**Table:**

	*U*^11^	*U*^22^	*U*^33^	*U*^12^	*U*^13^	*U*^23^
O1	0.0371 (8)	0.0425 (8)	0.0621 (10)	0.0207 (7)	0.0328 (7)	0.0302 (8)
O2	0.0361 (8)	0.0331 (7)	0.0443 (8)	0.0126 (6)	0.0193 (6)	0.0256 (7)
N1	0.0263 (8)	0.0247 (7)	0.0326 (8)	0.0101 (6)	0.0141 (6)	0.0145 (6)
C1	0.0302 (10)	0.0397 (11)	0.0618 (14)	0.0153 (9)	0.0227 (10)	0.0337 (11)
C2	0.0300 (10)	0.0315 (10)	0.0448 (11)	0.0132 (8)	0.0193 (9)	0.0236 (9)
C3	0.0254 (9)	0.0245 (8)	0.0262 (9)	0.0074 (7)	0.0123 (7)	0.0123 (7)
C4	0.0241 (8)	0.0263 (9)	0.0282 (9)	0.0089 (7)	0.0139 (7)	0.0134 (7)
C5	0.0261 (9)	0.0228 (8)	0.0234 (8)	0.0082 (7)	0.0106 (7)	0.0092 (7)
C6	0.0298 (9)	0.0242 (9)	0.0324 (9)	0.0073 (7)	0.0160 (8)	0.0150 (8)
C7	0.0269 (9)	0.0290 (9)	0.0350 (10)	0.0095 (7)	0.0174 (8)	0.0152 (8)
C8	0.0247 (9)	0.0234 (8)	0.0240 (8)	0.0091 (7)	0.0112 (7)	0.0094 (7)
C9	0.0280 (9)	0.0282 (9)	0.0300 (9)	0.0120 (7)	0.0137 (8)	0.0097 (8)
C10	0.0338 (10)	0.0345 (10)	0.0415 (11)	0.0183 (8)	0.0179 (9)	0.0168 (9)
C11	0.0277 (9)	0.0208 (8)	0.0253 (9)	0.0056 (7)	0.0091 (7)	0.0094 (7)
C12	0.0346 (10)	0.0273 (9)	0.0390 (10)	0.0151 (8)	0.0197 (8)	0.0195 (8)
C13	0.058 (7)	0.049 (6)	0.073 (7)	0.007 (4)	0.035 (5)	0.034 (5)
Cl1	0.0466 (10)	0.0642 (12)	0.0263 (6)	0.0269 (8)	0.0168 (6)	0.0158 (6)
C13B	0.091 (12)	0.052 (7)	0.118 (12)	0.036 (7)	0.066 (9)	0.060 (8)
Cl1B	0.0366 (17)	0.0457 (16)	0.0344 (14)	0.0273 (14)	0.0139 (11)	0.0231 (12)

### Geometric parameters (Å, °)

**Table:**

O1—C9	1.225 (2)	C7—C8	1.393 (3)
O2—C11	1.216 (2)	C7—H7	0.9500
N1—C9	1.362 (2)	C9—C10	1.504 (3)
N1—C8	1.408 (2)	C10—H10A	0.9800
N1—C1	1.482 (2)	C10—H10B	0.9800
C1—C2	1.525 (3)	C10—H10C	0.9800
C1—H1A	0.9900	C11—C12	1.525 (3)
C1—H1B	0.9900	C12—C13	1.598 (10)
C2—C3	1.509 (2)	C12—C13B	1.641 (13)
C2—H2A	0.9900	C12—Cl1B	1.689 (3)
C2—H2B	0.9900	C12—Cl1	1.736 (3)
C3—C4	1.380 (2)	C12—H12	0.9997
C3—C8	1.398 (2)	C13—H13A	0.9800
C4—C5	1.402 (2)	C13—H13B	0.9800
C4—H4	0.9500	C13—H13C	0.9800
C5—C6	1.395 (3)	C13B—H13D	0.9800
C5—C11	1.487 (2)	C13B—H13E	0.9800
C6—C7	1.385 (3)	C13B—H13F	0.9800
C6—H6	0.9500		
			
C9—N1—C8	126.44 (15)	N1—C9—C10	116.09 (17)
C9—N1—C1	123.37 (15)	C9—C10—H10A	109.5
C8—N1—C1	110.18 (14)	C9—C10—H10B	109.5
N1—C1—C2	105.33 (15)	H10A—C10—H10B	109.5
N1—C1—H1A	110.7	C9—C10—H10C	109.5
C2—C1—H1A	110.7	H10A—C10—H10C	109.5
N1—C1—H1B	110.7	H10B—C10—H10C	109.5
C2—C1—H1B	110.7	O2—C11—C5	121.51 (17)
H1A—C1—H1B	108.8	O2—C11—C12	119.99 (16)
C3—C2—C1	104.15 (15)	C5—C11—C12	118.47 (15)
C3—C2—H2A	110.9	C11—C12—C13	112.2 (5)
C1—C2—H2A	110.9	C11—C12—C13B	106.7 (7)
C3—C2—H2B	110.9	C13—C12—C13B	112.7 (8)
C1—C2—H2B	110.9	C11—C12—Cl1B	112.0 (2)
H2A—C2—H2B	108.9	C13—C12—Cl1B	2.8 (6)
C4—C3—C8	120.43 (16)	C13B—C12—Cl1B	110.3 (6)
C4—C3—C2	129.55 (16)	C11—C12—Cl1	108.14 (15)
C8—C3—C2	109.99 (15)	C13—C12—Cl1	109.6 (5)
C3—C4—C5	119.36 (16)	C13B—C12—Cl1	3.1 (7)
C3—C4—H4	120.3	Cl1B—C12—Cl1	107.28 (18)
C5—C4—H4	120.3	C11—C12—H12	109.1
C6—C5—C4	119.17 (16)	C13—C12—H12	108.7
C6—C5—C11	117.85 (16)	C13B—C12—H12	107.3
C4—C5—C11	122.97 (16)	Cl1B—C12—H12	111.2
C7—C6—C5	122.22 (16)	Cl1—C12—H12	109.0
C7—C6—H6	118.9	C12—C13—H13A	109.5
C5—C6—H6	118.9	C12—C13—H13B	109.5
C6—C7—C8	117.69 (17)	C12—C13—H13C	109.5
C6—C7—H7	121.2	C12—C13B—H13D	109.5
C8—C7—H7	121.2	C12—C13B—H13E	109.5
C7—C8—C3	121.11 (16)	H13D—C13B—H13E	109.5
C7—C8—N1	129.13 (16)	C12—C13B—H13F	109.5
C3—C8—N1	109.75 (15)	H13D—C13B—H13F	109.5
O1—C9—N1	121.97 (17)	H13E—C13B—H13F	109.5
O1—C9—C10	121.94 (17)		
			
C9—N1—C1—C2	−172.04 (17)	C9—N1—C8—C3	175.76 (17)
C8—N1—C1—C2	7.0 (2)	C1—N1—C8—C3	−3.2 (2)
N1—C1—C2—C3	−7.7 (2)	C8—N1—C9—O1	1.8 (3)
C1—C2—C3—C4	−175.90 (19)	C1—N1—C9—O1	−179.33 (19)
C1—C2—C3—C8	6.2 (2)	C8—N1—C9—C10	−177.82 (17)
C8—C3—C4—C5	1.6 (3)	C1—N1—C9—C10	1.1 (3)
C2—C3—C4—C5	−176.09 (18)	C6—C5—C11—O2	12.3 (3)
C3—C4—C5—C6	−1.0 (3)	C4—C5—C11—O2	−166.21 (17)
C3—C4—C5—C11	177.50 (16)	C6—C5—C11—C12	−165.37 (16)
C4—C5—C6—C7	−0.2 (3)	C4—C5—C11—C12	16.1 (3)
C11—C5—C6—C7	−178.82 (17)	O2—C11—C12—C13	25.4 (6)
C5—C6—C7—C8	0.8 (3)	C5—C11—C12—C13	−156.9 (5)
C6—C7—C8—C3	−0.2 (3)	O2—C11—C12—C13B	−98.5 (6)
C6—C7—C8—N1	178.75 (17)	C5—C11—C12—C13B	79.3 (6)
C4—C3—C8—C7	−1.0 (3)	O2—C11—C12—Cl1B	22.4 (3)
C2—C3—C8—C7	177.12 (17)	C5—C11—C12—Cl1B	−159.9 (2)
C4—C3—C8—N1	179.83 (16)	O2—C11—C12—Cl1	−95.6 (2)
C2—C3—C8—N1	−2.0 (2)	C5—C11—C12—Cl1	82.12 (19)
C9—N1—C8—C7	−3.3 (3)	C5—C11—C12—Cl1	82.12 (19)
C1—N1—C8—C7	177.69 (19)	C5—C11—C12—Cl1B	−159.9 (2)

### Hydrogen-bond geometry (Å, °)

**Table:**

*D*—H···*A*	*D*—H	H···*A*	*D*···*A*	*D*—H···*A*
C1—H1B···O2^i^	0.99	2.44	3.252 (3)	139
C4—H4···O1^ii^	0.95	2.48	3.430 (2)	177
C12—H12···O1^ii^	1.00	2.41	3.318 (2)	151

Symmetry codes: (i) *x*, *y*−1, *z*; (ii) *x*−1, *y*, *z*.

### Table 2 π-π stacking between symmetry-related phenyl rings. [Symmetry code: (iii) 1-x, -y, -z]

**Table:**

	Centroid–Centroid (Å)	Centroid-to-plane (Å)	Slippage (Å)
Cg1···Cg1^iii^	3.6959 (9)	3.4713 (6)	1.269
